# Anesthetic management during a cesarean section in a patient with cleidocranial dysplasia: a case report

**DOI:** 10.1186/s40981-017-0141-2

**Published:** 2018-01-05

**Authors:** Yumiko Nishio, Teruyuki Hiraki, Hiroko Taniguchi, Kazuo Ushijima

**Affiliations:** Department of Anesthesiology, Kurume University School of Medicine, 67 Asahi-machi, Kurume, Fukuoka 830-0011 Japan

**Keywords:** Cleidocranial dysplasia, Cesarean section, Neuraxial anesthesia

## Abstract

**Background:**

Cleidocranial dysplasia is a type of skeletal dysplasia, which is primarily characterized by delayed ossification of skeletal structures. It causes facial and oral abnormalities, resulting in difficult airway management and neuraxial anesthesia.

**Case presentation:**

The patient was a 24-year-old primipara (height 138 cm, weight 42 kg) with a hypoplastic right clavicle, patent fontanelles, dental malalignment, and a high palate. She was diagnosed with cleidocranial dysplasia at birth, although gene examination has not been performed. The fetus was confirmed to have short limbs and large fontanelles during an examination performed at 28 weeks gestation, suspected to have cleidocranial dysplasia. The mother was scheduled for a cesarean section at 37 weeks and 1 day due to cephalopelvic disproportion. Preoperative radiography and magnetic resonance imaging revealed no vertebral and spinal abnormalities, which allowed combined spinal-epidural analgesia (CSEA) to be performed. The surgery was safely concluded under CSEA with no intraoperative respiratory or circulatory problems.

**Conclusions:**

Patients with cleidocranial dysplasia exhibit facial, oral abnormalities, and often vertebral abnormalities. Imaging assessments before neuraxial anesthesia and careful preparation for airway management are required.

## Background

Cleidocranial dysplasia (CCD) is a type of skeletal dysplasia, which is characterized by hypoplastic clavicles, delayed closure of the fontanelles, delayed eruption of the second dentition, and a short stature. The prevalence of CCD is 1/1,000,000 [1], and there have only been few reports describing anesthesia in patients with CCD [2–4]. CCD patients have anatomical abnormalities that can pose problems during the management of general or neuraxial anesthesia. Here, we report a case with CCD who underwent cesarean section using combined spinal-epidural anesthesia (CSEA).

## Case presentation

A 24-year-old primipara (height 138 cm, weight 42 kg, non-pregnant weight 37 kg) was scheduled for cesarean section at 37 weeks and 1 day due to cephalopelvic disproportion. A diagnosis of CCD was made at birth, although gene examination has not been performed. She underwent tonsillectomy under general anesthesia in middle school and dilatation and curettage under sedation with intravenous anesthesia 2 years ago. Both of these procedures were performed uneventfully. At prenatal checkups conducted at 28 weeks gestation, the fetus also had short limbs and cranial defects, suggesting both the patient and the fetus were suspected of having skeletal dysplasia. Preanesthetic examination demonstrated a hypoplastic right clavicle, patent fontanelles, dental malalignment, and a high palate. Because thoracoabdominal radiography and magnetic resonance imaging (MRI) revealed no abnormalities in the spinal cord or vertebra, spinal and continuous epidural anesthesia was planned for cesarean section and postoperative analgesia.

After inserting an epidural catheter from the L1/2 intervertebral space, 1.5 mL of 0.5% hyperbaric bupivacaine was injected via the L3/4 intervertebral space for spinal anesthesia. Because the highest level of sensory blockade was the L1, 6 mL of 2% mepivacaine was administered from the epidural catheter, which extended analgesia to the T4 level bilaterally. Cesarean section was completed uneventfully, and a newborn weighing 2394 g with Apgar scores of 8 and 9 at 1 and 5 min, respectively, was delivered. The total amount of intraoperative blood loss, including amniotic fluid, was 2186 g. The operation and anesthesia lasted 59 and 83 min, respectively. The patient was discharged without any complications. The child was diagnosed with CCD based on their postnatal radiography and clinical findings.

### Discussion

CCD is a systemic bone disease, which is characterized by delayed ossification of skeletal structures, hypoplastic clavicles, delayed closure of the fontanelles, delayed eruption of the second dentition, and a short stature. Its hereditary form is inherited in an autosomal dominant manner, and the causative gene is located on chromosome 6p21, which encodes runt-related transcription factor 2 (one of the transcription factors in the runt domain-containing gene family) [5].

Patients with CCD exhibit various features, including a high palate, dental malalignment, and micrognathia as well as spinal or vertebral abnormalities such as scoliosis or spondylosis. These anomalies make ventilation, intubation, and neuraxial anesthesia difficult. Imaging examinations should be performed before neuraxial anesthesia in patients with CCD [2–4]. We evaluated this patient using MRI before CSEA. Furthermore, we prepared the equipment required for the difficult airway management such as bronchofiberscope and supraglottic airway devices in case the CSEA had an insufficient effect and general anesthesia was required. Fiberoptic intubation in a patient with CCD was reported because direct laryngoscopy visualized only the posterior commissure [2]. Furthermore, thoracic hypoplasia due to the absence or underdevelopment of the clavicles or ribs can cause postoperative respiratory failure [3, 4]. As the present case did not have any abnormal findings in the spine, we could perform spinal-epidural anesthesia without any problems.

In pregnant women complicated with CCD, delayed ossification of the pubic bone causes symphysiolysis and a contracted pelvis, which often require cesarean section. The reported frequency of cesarean section in such women is 69% [6]. Although regional anesthesia is frequently selected for pregnant women with CCD, the anesthetic level of single-shot spinal anesthesia can be unpredictable due to their short stature and spinal abnormalities. In our case, single-shot spinal anesthesia failed to provide sufficient analgesia levels and epidural analgesia was required. Furthermore, if a newborn has CCD, careful monitoring of its airway and respiratory status are required immediately after delivery [7].

## Conclusions

Patients with CCD are frequently accompanied with facial and oral anomalies as well as spinal abnormalities. Preoperative imaging assessments before neuraxial anesthesia are required. Also, careful consideration is required before induction of general anesthesia due to thoracic hypoplasia.

## Abbreviations

CCD: Cleidocranial dysplasia
CSEA: Combined spinal-epidural anesthesia
MRI: Magnetic resonance imaging
